# An increase in adenosine-5’-triphosphate (ATP) content in rostral ventrolateral medulla is engaged in the high fructose diet-induced hypertension

**DOI:** 10.1186/1423-0127-21-8

**Published:** 2014-01-27

**Authors:** Kay LH Wu, Chun-Ying Hung, Julie YH Chan, Chih-Wei Wu

**Affiliations:** 1 Center for Translational Research in Biomedical Sciences, Chang Gung Memorial Hospital-Kaohsiung Medical Center, Kaohsiung 83301, Taiwan

**Keywords:** Adenosine-5’-triphosphate, Metabolic syndrome, Fructose, Fructolysis, Glucose transporter, Ketohexokinase, Rostral ventrolateral medulla, Hypertension

## Abstract

**Background:**

The increase in fructose ingestion has been linked to overdrive of sympathetic activity and hypertension associated with the metabolic syndrome. The premotor neurons for generation of sympathetic vasomotor activity reside in the rostral ventrolateral medulla (RVLM). Activation of RVLM results in sympathoexcitation and hypertension. Neurons in the central nervous system are able to utilize fructose as a carbon source of ATP production. We examined in this study whether fructose affects ATP content in RVLM and its significance in the increase in central sympathetic outflow and hypertension induced by the high fructose diet (HFD).

**Results:**

In normotensive rats fed with high fructose diet (HFD) for 12 weeks, there was a significant increase in tissue ATP content in RVLM, accompanied by the increases in the sympathetic vasomotor activity and blood pressure. These changes were blunted by intracisternal infusion of an ATP synthase inhibitor, oligomycin, to the HFD-fed animals. In the catecholaminergic-containing N2a cells, fructose dose-dependently upregulated the expressions of glucose transporter 2 and 5 (GluT2, 5) and the rate-limiting enzyme of fructolysis, ketohexokinase (KHK), leading to the increases in pyruvate and ATP production, as well as the release of the neurotransmitter, dopamine. These cellular events were significantly prevented after the gene knocking down by lentiviral transfection of small hairpin RNA against KHK.

**Conclusion:**

These results suggest that increases in ATP content in RVLM may be engaged in the augmented sympathetic vasomotor activity and hypertension associated with the metabolic syndrome induced by the HFD. At cellular level, the increase in pyruvate levels via fructolysis is involved in the fructose-induced ATP production and the release of neurotransmitter.

## Background

The shift of our eating habit to a Western style and consumption of food rich in fatty acid and fructose increase the risks of developing obesity, insulin resistance, metabolic syndrome and diabetes [1-3]. Large populations of patients with obese and metabolic syndrome manifest hypertension [4]. Accumulating evidence suggests that high fructose diet (HFD) leads to the development of human metabolic syndrome with complications of hypertension, hyperglycemia, hypertriglyceridemia and insulin resistance [2-5]. The increase in fructose uptake has been linked to overdrive of sympathetic activity [6,7], although the underlying mechanism is not fully understood.

Neurons depends on aerobic metabolism of glucose for supply of adenosine-5’-triphosphate (ATP). Fructose, an isomer of glucose in structure, is therefore not an ordinary fuel to neuron. However, fructose is able to generate pyruvate which enters the citric acid cycle as acetyl coenzyme A under aerobic conditions [8,9]. The acetyl coenzyme A can then produce energy via the mitochondrial respiratory chain. The metabolic pathway that converts fructose into pyruvate is known as fructolysis. The rate-limiting enzyme of fructolysis is ketohexokinase (KHK) which phosphorylates and converts fructose to fructose-1-phosphate for pyruvate generation. Genes that required for fructose metabolism are expressed in the neurons [10], indicating neurons in the brain are able to utilize fructose as a carbon source of ATP production. In hippocampal region, it was reported that energy generated via fructolysis sustains neuronal integrity in the absence of glucose [11,12].

The rostral ventrolateral medulla (RVLM) contains premotor neurons for generation of supraspinal sympathoexcitatory outflow [13,14], and therefore is an important neural substrate for tonic regulation of sympathetic nerve activity and cardiovascular functions [14]. It is well established that increases in neuronal activity in RVLM and the release of neurotransmitters and neuromodulators from the RVLM projecting neurons at sympathetic preganglionic neurons in the spinal cord are the major source of the augmented sympathetic nervous system associated with hypertension [15,16].

In the present study, we examined whether fructose serves as an energy substrate in the RVLM and the significance of alterations in ATP content at RVLM in pathogenesis of the metabolic syndrome-associated hypertension. In an *in vivo* animal model of metabolic syndrome induced by the HFD, we found that an increase in tissue ATP content in RVLM contributed to the augmented sympathetic vasomotor activity and the increase in blood pressure. We further examined the molecular mechanism underlying high fructose-promoted ATP production and regulation of cellular functions by the use of an *in vitro* Neuro 2A (N2a) cell line. We found that fructose upregulated glucose transporter 2 and 5 (GluT2, 5) and KHK expressions, leading to an increase in ATP production and the release of the neurotransmitter dopamine from the N2a cells. These results suggest that increases in ATP content in RVLM is engaged in the augmented sympathetic vasomotor activity and hypertension associated with metabolic syndrome induced by the HFD. At cellular level, ATP production via fructolysis is involved in the release of neurotransmitter.

## Methods

### Animals

Male, adult Wistar Kyoto (WKY) rats were purchased from the Experimental Animal Center, National Science Council, Taiwan. Animals were allowed to acclimatize in a temperature- (22 ± 1°C) and light- (12:12 light–dark cycle, light on from 0:800) controlled room in an AAALAC certified animal facility for at least 14 days before the experiments. All experiments were carried out in accordance to the guidelines for animal experimentation endorsed by our Institutional Animal Care and use committee. Animals were randomly divided into the normal diet (ND) and high fructose diet (HFD) groups. In the HFD group, animals received 60% fructose (TD.89247, Harlan Laboratories, Indianapolis, IN, USA) as sole food source. The ND animals received regular chow (Purina, St. Louis, MO, USA)*.* Both food and water were provided *ad libitum*. Body weight, as well as food and water intake of animals were measured and recorded twice a week.

### Measurement of blood pressure

Systemic arterial pressure (SAP) was measured non-invasively under conscious condition using the tail-cuff plethysmography (BP-2000, Visitech, Apex, NC, USA). Animals were trained for 1 week in a pre-warmed tail-cuff device to accustom the procedure, followed by measurements of SAP twice per week. During each measurement, SAP was recorded for 30 min between 13:00 and 15:00; and data were collected for at least 12 weeks. In each measurement, 10 readings were collected and the mean value of all readings was used for the SAP.

### Power spectral analysis of SAP signals

Power spectral analysis of SAP signals was performed in animals with sodium pentobarbital (50 mg·kg^-1^, i.p.) anesthesia. SAP signal was monitored for 30 min between 13:00 and 15:00 by femoral artery cannulation. Power spectral analysis of SAP signals was performed by fast Fourier transform. We focused on the low frequency (LF, 0.25-0.8 Hz) component of SAP spectrum, as it takes origin from the RVLM [17-19] and reflect the prevalence of sympathetic nerve activity to the vessels.

### Collection of tissue samples from RVLM

To obtain samples for biochemical and molecular analyses, the brain stem was rapidly removed and placed on ice. Tissues on both sides of the ventrolateral part of medulla oblongata, at the level of RVLM (0.5 to 1.5 mm rostral to the obex) were collected by micropunches made with a stainless steel bore (1 mm ID) [17,19]. The collected tissues were stored immediately in liquid nitrogen for further analyses.

### Total protein isolation

Tissue samples from RVLM were homogenized with a Dounce grinder with a tight pestle in ice-cold lysis buffer (15 mM HEPES, pH 7.2, 60 mM KCl, 10 mM NaCl, 15 mM MgCl_2,_ 250 mM Sucrose, 1 mM EGTA, 5 mM EDTA, 1 mM PMSF, 2 mM NaF, 4 mM Na_3_VO_4_). A mixture of leupeptin (8 μg/mL), aprotinin (10 μg/mL), phenylmethylsulfonyl fluoride (20 μg/mL) and trypsin inhibitor (10 μg/mL) was included in the isolation buffer to prevent protein degradation. The homogenate was centrifuged at 13,500 *rpm* for 15 minutes, and the supernatant was collected for protein analysis. The concentration of the total protein extracted was estimated by the method of Bradford with a protein assay kit (Bio-Rad, Hercules, CA, USA).

### Measurement of intracellular ATP concentration

Tissue ATP content was determined by ATP colorimetric assay kit (Biovision, Milpitas, CA, USA). Total protein samples from RVLM were centrifuged at 13500 *rpm* for 15 min, and 10 μl supernatant was incubated with ATP reaction mixture for 30 min. The ATP levels were detected at 570 nm using a microplate reader (Thermo Fisher Scientific Inc., Waltham, MA, USA). To determine cellular ATP level in the N2a cells, samples were collected in a lysis buffer (Thermo Fisher Scientific Inc.) and centrifuged at 13500 *rpm* for 15 min. Supernatant (10 μl) was incubated with ATP reaction mixture for 30 min and detected by the same procedures. ATP levels were normalized to the protein concentration of the samples. Protein concentrations were determined by the Bradford analysis. All experiments were repeated in quadruplicates.

### Measurement of intracellular pyruvate concentration

Cellular pyruvate concentration was determined by the pyruvate assay kit (Biovision). In brief, 50 μg lysate from each sample was incubated with pyruvate reaction mixture in a light-proof condition for 30 min. Pyruvate level was detected at 570 nm using a microplate reader (Thermo Fisher Scientific Inc.). Pyruvate concentration was normalized to the protein concentration of the samples. Protein concentrations were determined by the Bradford analysis. Each assay was performed in quadruplicates.

### Measurement of tissue level of the reactive oxygen species

Levels of reactive oxygen species (ROS) were measured by flow cytometry using the fluorescence dye dihydroethidium (DHE) for detection of the ROS. Tissue from RVLM was rinsed, cut into small pieces and sieved by a strainer (cut-off size of 100 μm). Separated cells were fixed with 4% paraformaldehyde for 10 min at room temperature, followed by incubation with DHE (5 μM, 0.15% DMSO) in a light proof condition at the room temperature. The level of cellular ROS was analyzed with Gallios™ (Beckman, CA, USA) flow cytometer. Each determination is based on mean fluorescence intensity of 5,000 cells.

### Western blot analysis

Proteins were separated by 10-12% SDS-PAGE and transferred onto PVDF membrane (Immobilon-P membrane; Millipore; Bedford, MA, USA). Membranes were incubated with specific antibodies, followed by washing and incubation with a horseradish peroxidase-conjugated secondary antibody. Specific antibody-antigen complex was detected using an enhanced chemiluminescence Western Blot detection system (Thermo Fisher Scientific Inc.). The amount of detected proteins was quantified by ImageJ software (NIH, Bethesda, MD, USA), and was expressed as the ratio to β-actin protein. Each assay was performed in quadruplicates.

### Intracisternal infusion of the test agent

After anesthetized with sodium pentobarbital (50 mg/kg, IP), a midline dorsal neck incision was made, and the dura mater between the foramen magnum and C1 lamina was exposed following dissection of the overlying muscles. The dura was perforated with a 22-gauge steel needle. After observation of leakage of cerebrospinal fluid (CSF) from this hole, a PE-10 catheter (Clay Adams, Sparks, MD, USA) was advanced for 3 mm into the cisterna magna. The infusion catheter was sealed to the dura with tissue glue and the incision was closed with layered sutures. The outer end of the catheter was connected to an osmotic minipump (Alzet 1002; Durect Co., Cupertino, CA, USA), which was placed under the skin in the neck region [20,21]. Intracisternal (IC) infusion of oligomycin (70 fmol/μl/day) was carried out for 14 days. Infusion dose of oligomycin was determined in our pilot study. Control infusion of artificial CSF (aCSF) served as the volume and vehicle control. All animals received intramuscular injection of procaine penicillin (1,000 IU) postoperatively, and only those that showed progressive weight gain after the operation were used in the subsequent experiments.

### Cell culture

N2a cells was originally obtained from American Type Culture Collection (ATCC, Manassas, VA, USA) and was maintained in Eagle’s minimal essential medium (MEM; Invitrogen, Carlsbad, CA, USA) supplemented with 10% FBS (Invitrogen) plus 1% penicillin/streptomycin (Invitrogen) in an incubation chamber with 5% CO_2_ and 95% air at 37°C. One day before fructose application, cells were seeded onto a 10-cm dish at a density of 1 × 10^6^ cells per well. Different concentrations of fructose (0, 12.5, 25, or 50 μM) were applied to MEM solution. Following fructose application, the cells were incubated for 72 h before various analyses. MEM solution was replaced every two days to stable nutrition supply.

### MTS assays for cell viability

3-(4,5-dimethylthiazol-2-yl)-5-(3-carboxymethoxyphenyl)-2-(4-sulfophenyl)-2H-tetrazolium (MTS) assay (Promega, Madison, WI, USA) was used to quantify cell viability. Cells (100 μL, 1 × 10^5^ cells/ml) were seeded into a 96-well flat-bottomed plate at 37°C with 5% CO_2_. At 72 h after fructose treatment, the cells were washed with PBS for three times and replaced with fresh medium (100 μL). MTS (20 μL) reagents were added to each well, and the plate was incubated for 4 h under dark. Absorbance was recorded at 490 nm using a microplate reader (Thermo Fisher Scientific Inc.). Blank wells with no reagent were measured for luminescence and were deducted from the values of the experimental wells. Values of viability of the treated-cells were expressed as a percentage of that from corresponding control cells. All experiments were repeated in quadruplicates.

### Measurement of dopamine concentration

Dopamine level was measured by enzyme-linked immunosorbent assay kit (LDN^®^, Nordhorn, Germany). Medium of N2a cells was collected at 72 h after exposure to fructose alone or with additional drug treatment. 50 μl samples or standard were incubated with Detection Reagent A for 60 min at 37°C, followed by incubation with Detection Reagent B for 30 min at 37°C under light-proof condition. After 15–25 min substrate incubation, the reaction was stopped by Stop Solution. Dopamine level was detected at 450 nm using a microplate reader (Thermo Fisher Scientific Inc.) and was expressed as pg/ml of medium. All experiments were repeated in quadruplicates.

### Glucose uptake assay

A non-radioactive 2-deoxyglucose (2DG, 4 mmol/L) (Biovision) was incubated with N2a cells for 30 min after overnight starvation. Cells were then collected for homogenization and detection of intracellular metabolite, 2-DG6-phosphate (2-DG6P). Cell lysate was reacted with reagent A followed by reagent B. After incubation, the level of 2-DG6P was analyzed by the colorimetric enzymatic methods according to the manufacturer’s instructions. Each assay was performed in quadruplicates.

### Gene silencing of Ketohexokinase

The short hairpin RNA (shRNA) oligonucleotides for silencing KHK expression in N2a cell was purchased from Santa Cruz Biotechnology, Inc. (Santa Cruz, CA, USA). N2a cells were seeded into a 12-well Plate 24 h prior to lentiviral infection. 1 mL medium (with serum and antibiotics) was added and incubated with cells overnight. On the day of infection, the culture media was removed from the wells and replaced with 1 mL of Polybrene/media mixture. Lentiviral particles (1 × 10^5^ plague forming units/1 mL) were thawed at room temperature and mix gently and incubated with cells overnight. This was followed by removal of the culture medium and replaced with 1 mL of complete medium without Polybrene overnight. To select stable clones expressing the shRNA, cells were split in 1:3 and incubated continuously for 24–48 h in the medium. Stable clones expressing the shRNA were selected by Puromycin dihydrochloride, and were followed by replacing the medium with fresh puromycin-containing medium every 3–4 days until resistant colonies were identified. These colonies were expanded and assayed for stable shRNA expression. Cells were transfected in parallel with control shRNA lentiviral particles (Santa Cruz Biotechnology) as negative controls.

### Immunofluorescence staining

The cell was fixed 15 min in the 4% paraformaldehyde in 0.1 M phosphate buffered saline (PBS, pH 7.4). After washed with PBS and permeabilization with 0.1% Triton X 100 in 0.1% sodium citrate, the cell was incubated with the anti-KHK antibody at room temperature for 2 hour and then rinsed 3 times in PBS. After incubation in fluorescence conjugated IgG (Vector Laboratories, Burlingame, CA, USA) for 1 hour, the cells were rinsed with PBS. 4’,6-diamidino-2-phenylindole (DAPI) was employed to identify the morphology of cell nuclei. The expression and distribution of fluorescence signals were detected under an Olympus Fluoview 1000 laser confocal microscope (Tokyo, Japan) equipped with a krypton/argon laser.

### RNA isolation and reverse transcription real-time polymerase chain reaction

To detect KHK mRNA expressions, total RNA from the treated cells were isolated with TRIzol reagent (Invitrogen, Carlsbad, CA, USA) according to the manufacturer’s protocol. All RNAs isolated were quantified by spectrophotometry and the optical density 260/280 nm ratio was determined. Reverse transcriptase (RT) reaction was performed using a SuperScript Preamplification System (Invitrogen) for the first-strand cDNA synthesis. Real-time PCR analysis was performed by amplification of cDNA using a LightCycler^®^ 480 instrument (Roche Diagnostics, Mannheim, Germany). The primers used in real-time PCR amplification were designed using Roche LightCycler probe design software 2.0 based on sequence information from the NCBI database, and were synthesized by Genemed Biotechnologies (San Francisco, CA, USA). The primer pairs for amplification of KHK cDNA are: forward (5′- GATACCCCTTGCTCTTGCTG-3′) and reverse (5′- TGCAGCATCTTCACCTGTTC-3′); β-actin cDNA are: forward (5′- GCTGAGAGGGAAATCGT -3′) and reverse (5′- CGTCAGGCAGCTCATAG -3′).

PCR reaction for each sample was carried out in duplicates for all the cDNA and for the β-actin control. The amplification protocol for cDNA on the LightCycler^®^ 480 was a 10 min denaturation step at 95°C for polymerase activation, a “touch down” PCR step of 10 cycles consisting of 10 s at 95°C, 10 s at 65°C, and 30 s at 72°C, followed by 40 cycles consisting of 10 s at 95°C, 10 s at 55°C, and 30 s at 72°C. After slow heating (0.1°C per second) the amplified product from 65°C to 95°C to generate a melting temperature curve, which serves as a specificity control, the PCR samples were cooled to 4°C. Fluorescence signals from the amplified products were quantitatively assessed using the LightCycler^®^ 480 software program. Second derivative maximum mode is chosen with baseline adjustment set in the arithmetic mode. The relative change in mRNA expression was determined by the fold-change analysis, in which Fold change = 2^-[ΔΔCt]^, where ΔΔCt = [(Ct_target__mRNA_ - Ct_β-actin_) _treatment_ - (Ct_target__mRNA_ - Ct_β-actin_)_control_]. Note that Ct value was the cycle number at which fluorescence signal crosses the threshold.

### Statistic analysis

Data are expressed as means ± SEM. The statistical software GraphPad Prism (La Jolla, CA, USA) was used for data analysis. For biochemical experiments that involved two groups of samples, Student’s unpaired *t* test was used. For biochemical experiments that involved multiple groups, one-way or two-way analysis of variance with repeated measures were used to assess group means. This was followed by the Tukey’s multiple range test for post hoc assessment of individual means. *P < 0.05 was considered statistically significant.

## Results

### High fructose diet increases blood pressure and sympathetic vasomotor tone

**Figure 1:**
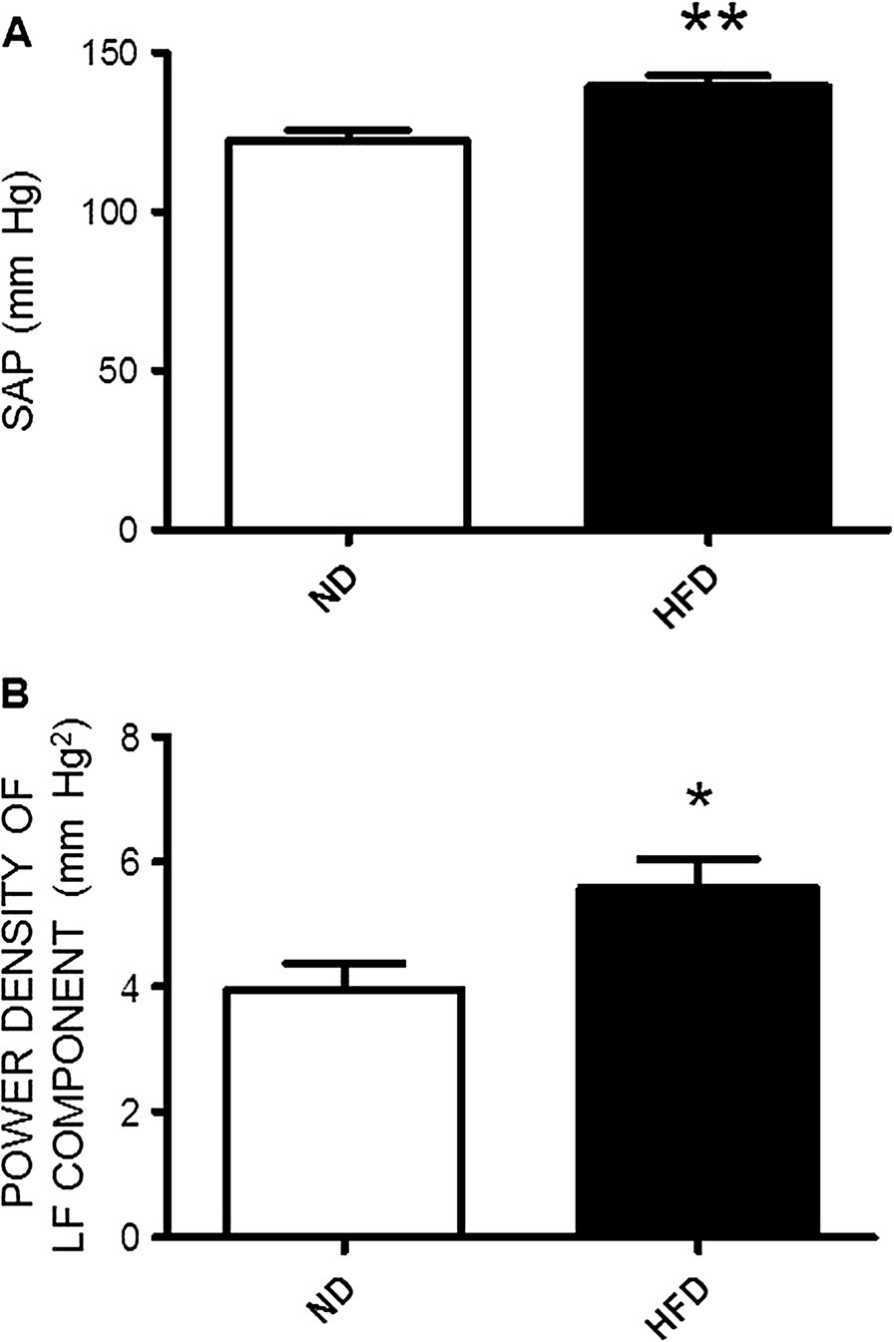
**High fructose diet induces increases in blood pressure and sympathetic vasomotor activity in rats.** The systemic arterial pressure (SAP, **A**) and power density of low frequency (LF, **B**) component of the SAP signals, our experimental index of the sympathetic vasomotor activity, measured in week 12 in animals subjected to the normal diet (ND) or high fructose diet (HFD). Values are mean ± SEM (n = 8 to 12 animals in each experimental group). *P < 0.05, **P < 0.01 versus ND group in the student *t* test.

In comparison to the ND group, animals received HFD for 12 weeks induced a significant increase in SAP (Figure 1A), accompanied by an increase in the sympathetic vasomotor activity reflected by the increase in power density of the LF component of SAP spectrum (Figures 1B). In addition, animals subject to the 12-weeks HFD developed characteristics of metabolic syndrome (Table 1). Feeding animals the HFD for 12 weeks, on the other hand, did not affect daily food intake (ND vs. HFD, 35.2 ± 1.23 g vs. 40.8 ± 2.43 g, n = 32, P > 0.05) or the body weight (ND vs. HFD, 340.3 ± 6.09 g vs. 341.8 ± 6.78 g, n = 32, P > 0.05) measured at the age of 17-weeks old.

**Table 1:** Metabolic parameters evaluated in week 12 after subjected the animals to the ND or HFD

	**ND (n = 12)**	**HFD (n = 12)**
Fasting blood sugar (mg/dL)	85.83 ± 1.88	93.08 ± 2.45*
Fasting blood insulin (pmol/L)	49.95 ± 5.98	168.70 ± 45.72*
HOMA-IR	0.92 ± 0.11	2.63 ± 0.59*
Fasting blood triglyceride (mmol/L)	113.40 ± 2.04	146.30 ± 12.81*
Waistline (cm)	15.98 ± 0.20	15.81 ± 0.21

Data are means ± SEM. *P < 0.05 vs. ND rats.

### High fructose diet increases tissue ATP content, KHK and glucose transporter expression in RVLM

**Figure 2:**
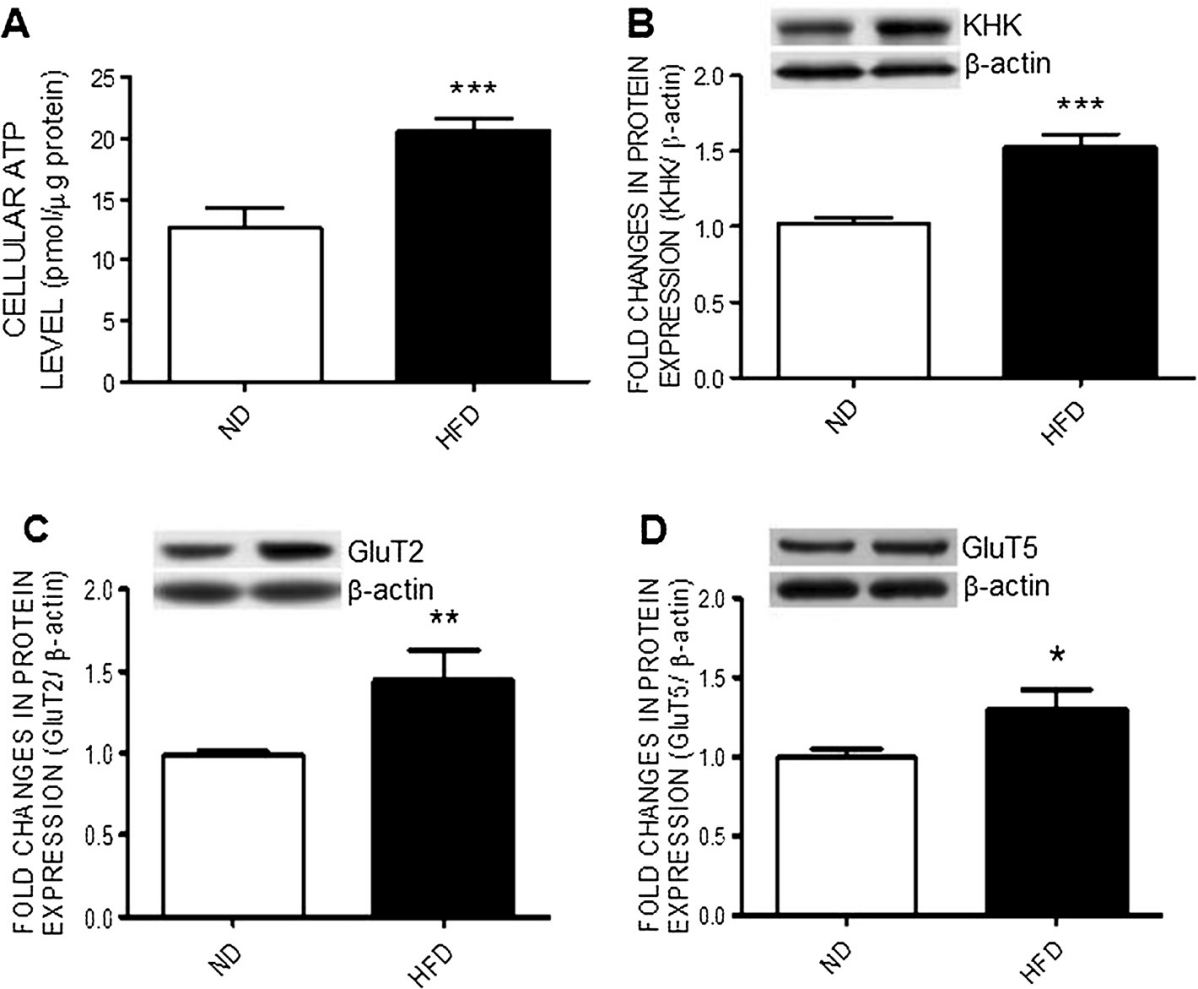
**High fructose diet increases tissue ATP content and upregulates ketohexokinase and glucose transporter expression in RVLM.** Tissue levels of ATP **(A)** and representative gels (inset) or densitometric analysis of protein level of ketohexokinase (KHK) **(B)** or glucose transporter 2 or 5 (GluT2 or GluT5) **(C and D)** in RVLM detected in week 12 after the ND or HFD. Values are mean ± SEM (n = 8 to 12 animals in each experimental group). *P < 0.05, **P < 0.01, ***P < 0.001 versus ND group in the student *t* test.

At the end of 12-weeks HFD, there was a significant increase in tissue ATP content in RVLM (Figure 2A). This is associated with upregulations in protein expressions of the rate-limiting enzyme of fructolysis, KHK, (Figure 2B) and the glucose transporters, GluT2 and GluT5, (Figure 2C) in the same brain region. For the negative control, we found that tissue ATP content in the hippocampus, a brain area that is not directly involved in the autonomic regulation of the blood pressure, was not affected (Additional file 1: Figure S1).

### High fructose diet upregulates protein expression of the mitochondrial electron transport chain enzyme complex in RVLM

**Figure 3:**
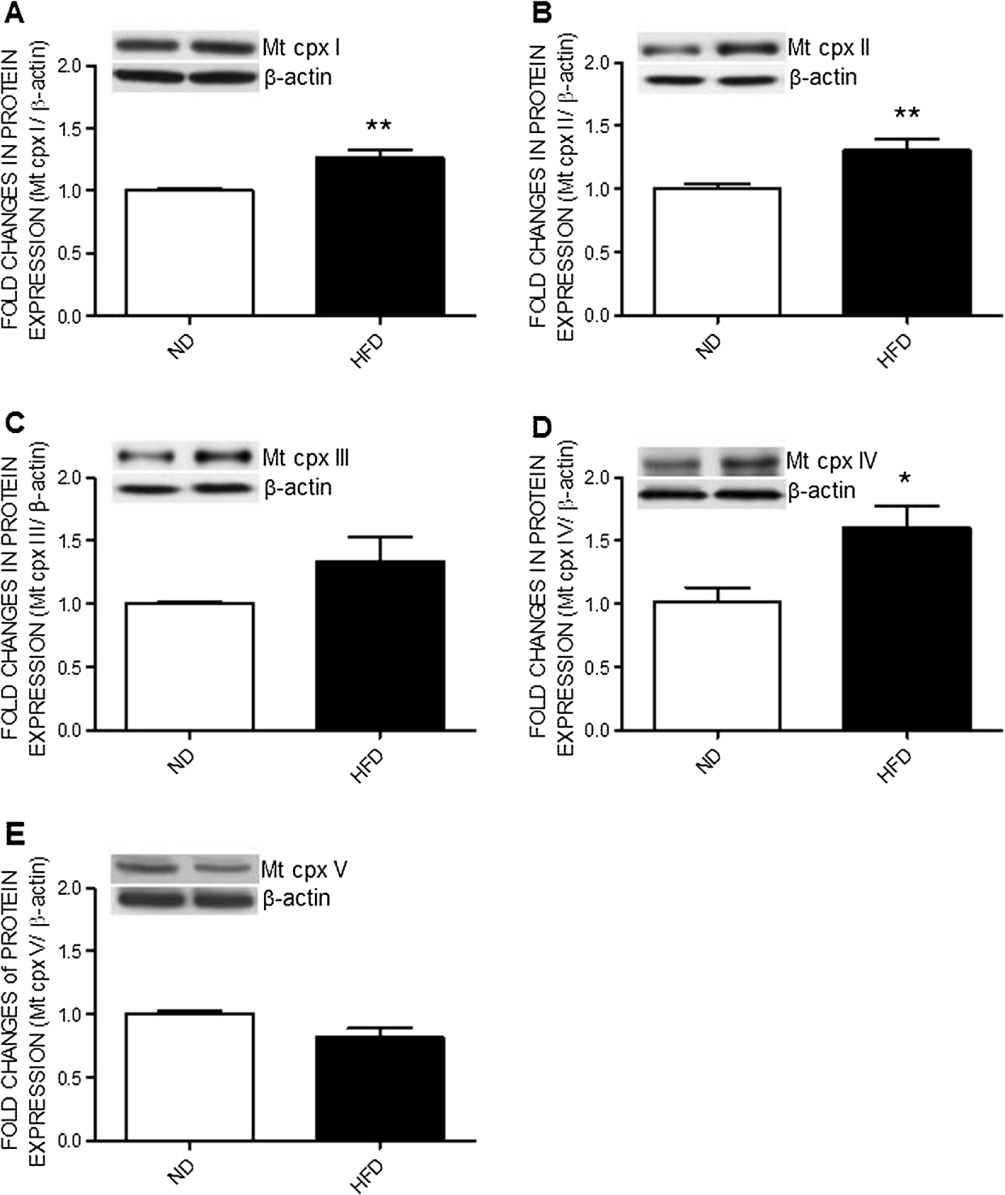
**High fructose diet induces upregulation of the mitochondrial respiratory chain enzymes in RVLM.** Representative gels (inset) and densitometric analysis of results from Western blot showing changes in expression of mitochondrial respiratory complex (Mt cpx) I to V **(A to E)** in week 12 after the ND or HFD. Values are mean ± SEM (n = 8 to 12 animals in each experimental group). *P < 0.05, **P < 0.01 versus ND group in the student *t* test.

To delineate whether the increase in tissue ATP level in RVLM resultant from the increase of oxidative phosphorylation, expression of mitochondrial respiratory chain proteins were determined. Compared with the ND-fed rats, the expression of mitochondrial respiratory complexes proteins, including Complexes I, II, and IV, were markedly increased in RVLM of the HFD-fed rats (Figure 3). Protein expressions of Complexes III and V in RVLM, on the other hand, were not affected by the HFD. The HFD also did not affect tissue level of the reactive oxygen species (ROS) (Additional file 2: Figure S2).

### Suppression of heightened ATP content in RVLM attenuates HFD-induced hypertension

**Figure 4:**
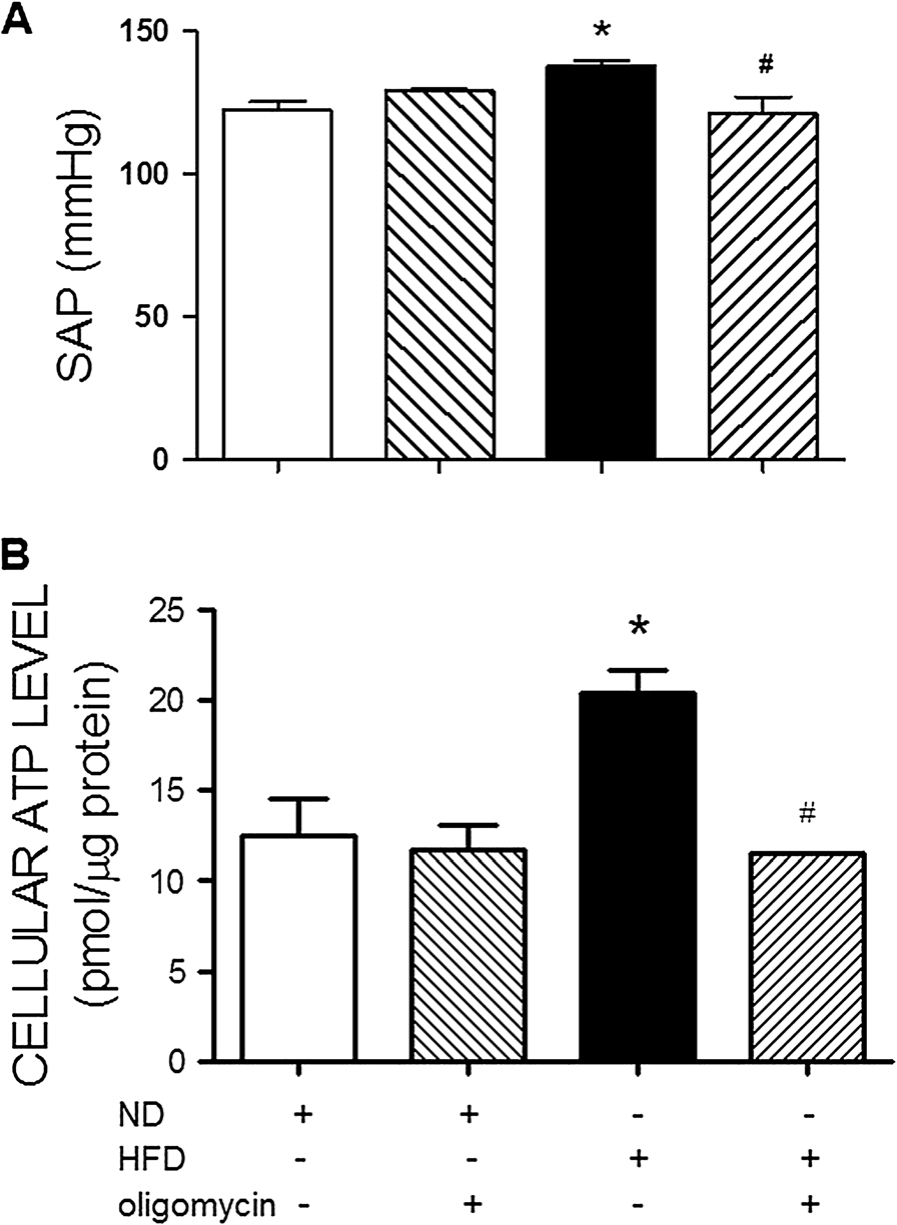
**Reversal by oligomycin on the HFD-induced pressor response and the increase in ATP content in RVLM.** The SAP **(A)** and tissue ATP content in RVLM **(B)** examined in week 12 after ND or HFD alone or with additional intracisternal infusion of oligomycin (70 fmol/μl/day for 14 days) from week 10 to 12. Values are mean ± SEM (n = 5 to 8 animals in each experimental group). *P < 0.05 versus ND group and ^#^*P* < 0.05 versus HFD group in the post hoc Tukey’s multiple range analysis.

To establish a causal role of ATP increment at RVLM in hypertension induced by the HFD, an ATP synthase inhibitor, oligomycin (70 fmol/μl/day), was infused into the cisterna magna from week 10 to 12 after the HFD. In comparison to aCSF infusion, IC infusion of oligomycin significantly blunted the pressor response in the HFD-fed animals (Figure 4A). The same treatment, however, did not affect the SAP in the ND-fed animals. IC infusion of oligomycin for 2 weeks prevented the increase in tissue level of ATP in RVLM of the HFD-fed animals. The same treatment also slightly suppressed ATP content in RVLM of the ND-fed animals (Figure 4B).

### Fructose increases ATP production and dopamine release from the catecholamine-containing N2a cells

**Figure 5:**
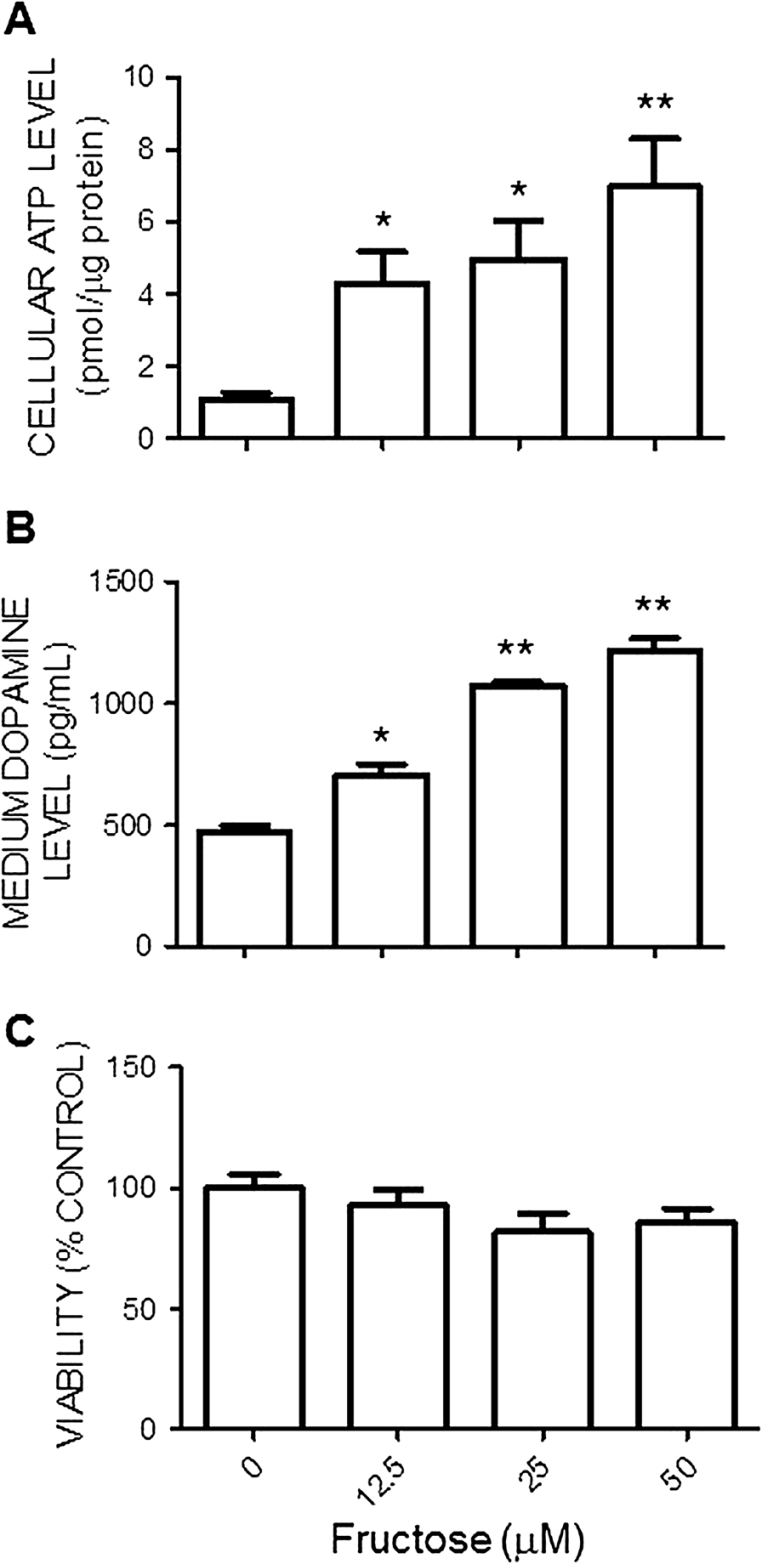
**Fructose increases cellular ATP levels and the release of dopamine from the Neuro 2a cells.** The cellular ATP contents **(A)**, levels of dopamine in culture medium **(B)** and cell viability **(C)** of the N2a cells determined at 72 hours after exposure to different concentrations of fructose (0, 12.5, 25, or 50 μM). Values are mean ± SEM of quadruplicate analyses. *P < 0.05, **P < 0.01 versus control (0 μM fructose) group in the post hoc Tukey’s multiple range analysis.

The N2a cell is a mouse neural crest-derived cell line that has been extensively used to study functions of the catecholamine-containing neurons [22]. This cell line was selected in the current study as an *in vitro* cell model to investigate mechanisms underlying the fructose-induced increase in ATP content and the effect of fructose on neuronal function, reflected by the release of neurotransmitter, dopamine. Incubation of the N2a cells with different concentrations of fructose (0, 12.5, 25, or 50 μM) in the culture medium for 72 h resulted in a dose-related increase in cellular ATP content (Figure 5A). Results from ELISA assay further showed that fructose caused a dose-dependent increase in dopamine content measured in the culture medium (Figure 5B). Under these dosing ranges, fructose had no notable effect on viability of the N2a cells (Figure 5C).

### Fructose upregulates ketohexokinase and glucose transporter expression as well as increases cellular pyruvate level in N2a cells

**Figure 6:**
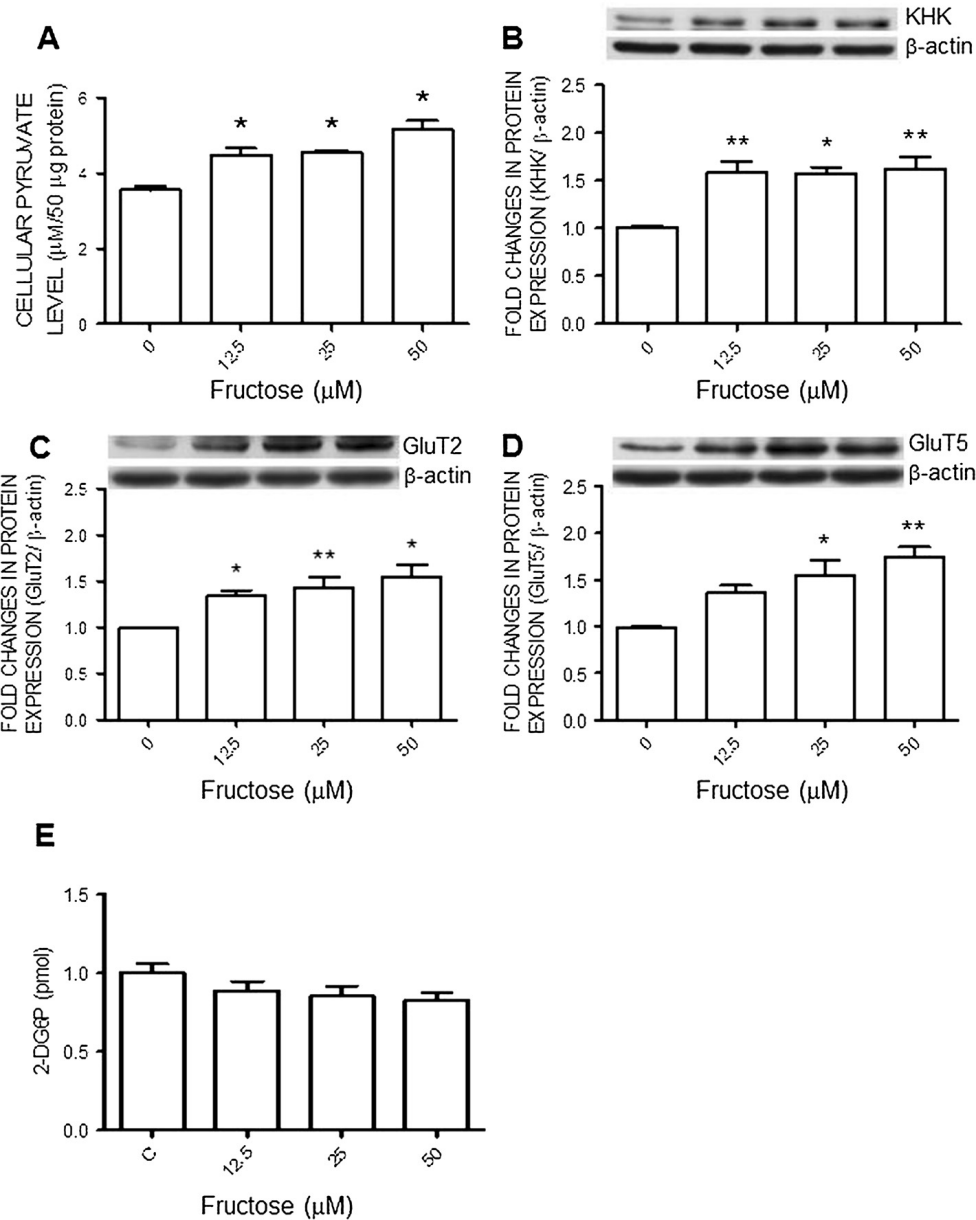
**Fructose upregulates the expression of ketohexokinase and glucose transporters in the N2A cells.** Cellular levels of pyruvate **(A)** and 2-deoxyglucose 6-phosphate (2-DG6P) **(E)**, or representative gels (inset) and densitometric analysis of results from Western blot showing changes in expression of KHK **(B)**, GluT2 **(C)** or GluT5 **(D)**, examined at 72 hours after the administration of different concentrations of fructose (0, 12.5, 25, or 50 μM) to the N2a cells. Values are mean ± SEM of quadruplicate analyses. *P < 0.05, **P < 0.01 versus control (0 μM fructose) group in the post hoc Tukey’s multiple range test.

Fructose is capable of generating pyruvate via fructolysis to be further used for energy generation. KHK is the rate-limiting enzyme in the fructolytic pathway. In the N2a cells we found that pyruvate levels were increased (Figure 6A) and KHK expression were significantly upregulated by the fructose in the culture medium (Figure 6B). The same treatments also upregulated protein expression of GluT2 and GluT5 (Figure 6C and D). In contrast, fructose had no effect on uptake of glucose to the N2a cells (Figure 6D).

### Ketohexokinase gene knocking down abolishes the fructose-induced pyruvate and ATP production as well as dopamine release

**Figure 7:**
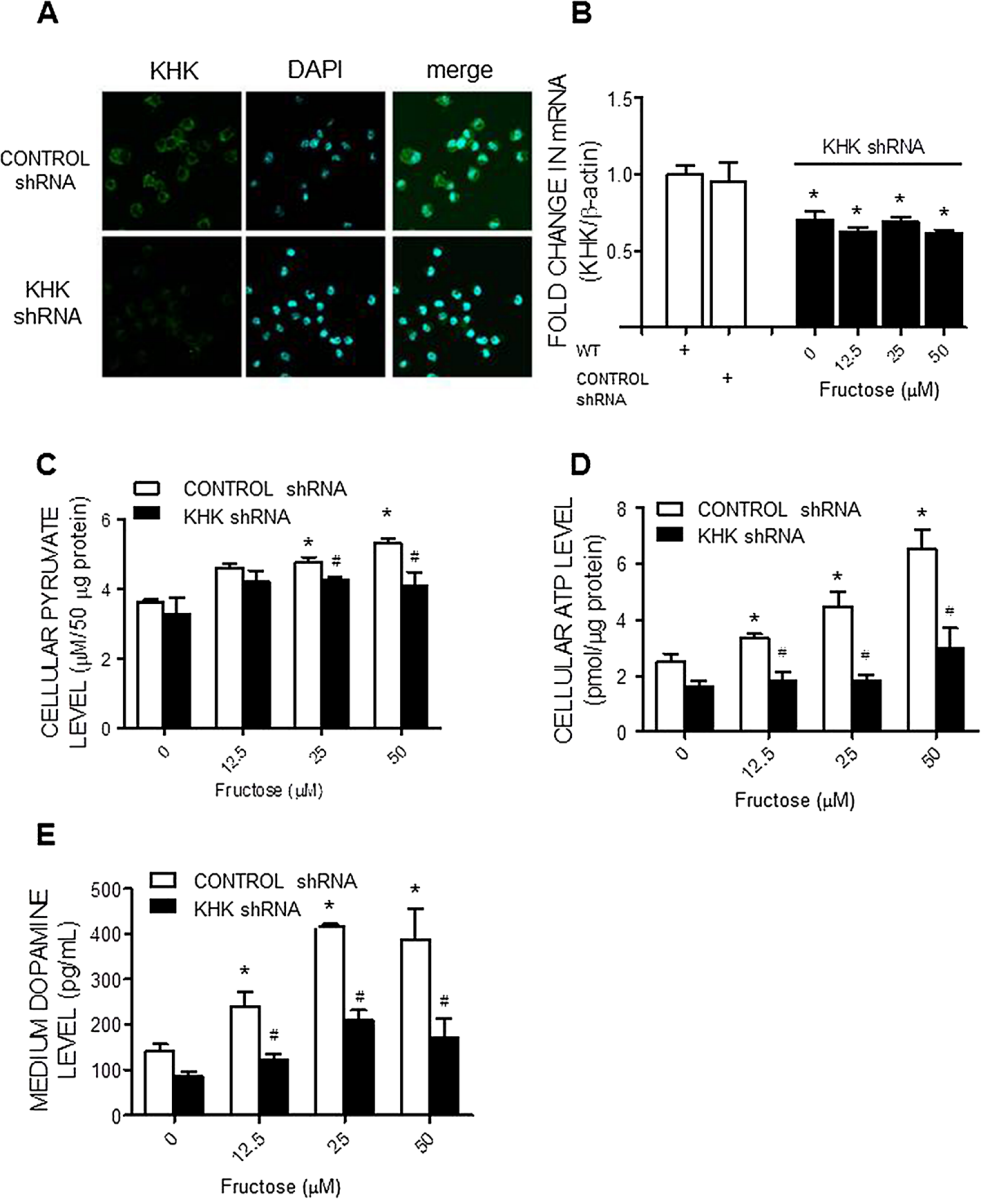
**KHK gene knockdown prevents the fructose-induced increases in cellular pyruvate and ATP level as well as dopamine release from the N2a cells.** Representative immunofluorescence images showing distribution of KHK in the N2a cells **(A)** on day 7 after the lentiviral transfection of small hairpin RNA of KHK (KHK shRNA) or control shRNA; as well as changes in KHK mRNA **(B)**, cellular pyruvate **(C)** and ATP contents **(D)**, and dopamine levels in the culture medium **(E)**, detected at 72 hours after the administration of different concentrations of fructose (0, 12.5, 25, or 50 μM) to the culture medium of the KHK shRNA or control shRNA-transfected N2a cells. Values are mean ± SEM of quadruplicate analyses. *P < 0.05 versus control (0 μM fructose) group and ^#^*P* < 0.05 versus CONTROL shRNA group in the post hoc Tukey’s multiple range tests. WT, wild type N2a cells. 4’,6-diamidino-2-phenylindole (DAPI) was used to identify nucleus in the cultured cells.

To further delineate a causal relationship of fructose-induced increases in KHK expression on ATP production and dopamine release, lentiviral transfection of KHK shRNA (0.5 MOI) was performed to knocking down KHK expression in the N2a cells. In comparison with lentiviral transfection of control shRNA, expression of KHK mRNA in the N2a cells was effectively suppressed at 72 h following the shRNA transfection (Figures 7A and B). Furthermore, when the KHK gene-silencing N2a cells was subjected to evaluation of pyruvate and ATP production at 72 h after incubation with fructose, the dose-related increases in their production by fructose were also significantly inhibited (Figures 7C and D), alongside a reversal of dopamine release (Figure 7E). Transfection of control shRNA had no effect on the fructose-induced increases in pyruvate and ATP production, or the dopamine release from the N2a cells.

## Discussion

The most significant finding of this study was an increase in tissue ATP content in RVLM and its engagement in the augmented sympathetic vasomotor activity and hypertension induced by the HFD. To our knowledge this is the first demonstration to link tissue ATP content in brain cardiovascular neurons to the altered central autonomic function in the setting of hypertension associated metabolic syndrome. At cellular level, our data from the N2a catecholaminergic cells further show that high fructose increases ATP production via upregulation of the transporters and enzyme that are involved in fructolysis for the generation of pyruvate. Functionally, the increased ATP plays an active role in the regulation of cellular phenotype by increasing the neurotransmitter release.

The metabolic syndrome is closely related to dysfunction of the autonomic nervous system. In this regard, patients with the syndrome are observed with an increased sympathetic nerve activity [23,24] and high urine or plasma catecholamine concentrations [25]. In the HFD-fed rats, an experimental model of human metabolic syndrome [26], we found in the current study that central sympathetic outflow, reflected by the increases in power density of the LF component of SAP spectrum [13], was significantly increased, alongside the pressor response. These findings extend the previous reports on the increase in peripheral sympathetic activity [27,28] and unravel an additional mechanism via activation of the central sympathetic outflow in manifestation of sympathetic overactivation and hypertension in the metabolic syndrome. In obesity-induced hypertension, a condition often observed in patients with metabolic syndrome, it was reported that the high blood pressure is related to sympathoexcitation in cardiovascular regulatory centers such as hypothalamic nuclei and the RVLM [29,30]. It is noteworthy that the LF component of the SAP signals originates from the RVLM [13]. Together these results suggest the RVLM as a major neural substrate for generation of sympathoexcitation and hypertension in the metabolic syndrome. Power density of the LF component of the SAP signals evaluated under anesthetic condition was comparable to that recorded under the conscious condition [31].We therefore reason that short term anesthesia might not affect the cardiovascular performance, at least the frequency components of the SAP spectrum. Normotensive rat fed with a HFD is a well-established rodent model for study of human metabolic syndrome. This model mimics several features, including hypertension, insulin resistance, and abnormal lipid profile, of the western diet-induced metabolic syndrome in patients [32,33]. Our results of no different in the waistline measurement are in consistence to the published data in which animals subjected to 60% HFD do not develop obesity [34]. As such the observations made in this study are not directly related to the obesity.

The mechanism linking the metabolic syndrome with central sympathoexcitation is not fully understood. We found that tissue ATP content was significantly increased in RVLM of the HFD-fed rats. An active role of tissue ATP in sympathoexcitation and hypertension in the metabolic syndrome was further established by the intervention to partially block ATP production in RVLM via IC infusion of the ATP synthase inhibitor, oligomycin. At a dose that only moderately inhibits ATP content in RVLM of the ND-fed rats, oligomycin prevents the increase in ATP content in RVLM following the HFD. This treatment also prevents the increase in central sympathetic outflow and hypertension. The sympathetic premotor neurons in RVLM are highly sensitive to ATP. They can be excited in response to exogenous ATP or ATP receptor agonists [35,36]. Moreover, an increase in tissue concentration of ATP in the RVLM leads to an increase in activity of the sympathetic premotor neurons, resulting in the increased central sympathetic drive [37]. Expression of the mitochondrial electron transport chain enzymes is essential for generation of ATP. Our results of upregulations of these enzyme complexes imply the induced increase in ATP content may attributed to the increase in the machinery of mitochondrial oxidative phosphorylation in RVLM. The observations that tissue level of ROS in the RVLM was not affected by the HFD further suggest a minor role of the radicals in neural mechanism of hypertension induced by the HFD. This finding is seemingly disparate to the previous reports of an active role of ROS in RVLM on neurogenic hypertension induced by angiotensin II [20] or obesity [29]. Although reasons behind the discrepancy are not immediately clear, it is possible that different signals in the RVLM may have their prevalence in mediating sympathoexcitation among different animal models of hypertension.

While the premotor neurons for generation of sympathetic vasomotor activity reside in the RVLM [13,14], integration of cardiovascular signals in the brain areas, such as the nucleus tractus solitarii (NTS) and hypothalamic paraventricular nucleus (PVN), are important for the autonomic regulation of the blood pressure. In our preliminary experiments, we found that there is no apparent difference in tissue ATP levels in the NTS or PVN of the ND versus HFD rats. In addition, in the hippocampus (a brain area that is not directly related to autonomic control of the blood pressure) of the HFD rats, there is no significant increase in tissue ATP content. Together these results indicate site specificity of the observations and imply that RVLM neurons are more sensitive to changes in tissue fructose level, although the involvement of other autonomic nuclei can not be excluded.

Fructose has been strongly linked to the increases in sympathetic activity associated with the metabolic syndrome [6,7]. The observations of an active role of ATP in RVLM on sympathoexcitation in the HFD-fed rats urged us to delineate cellular mechanisms underlying the fructose-induced ATP production. The N2a cell line has been used as a model for studying the function of the catecholamine-containing cells [22], of which the RVLM neurons belong to [13]. We found in N2a cells that fructose evoked a dose-related increase in pyruvate and ATP content both of which were blunted by the gene knocking down of KHK, the rate-limiting enzyme that converts fructose into pyruvate. Pyruvate enters the mitochondria where it is converted to Acetyl CoA for further generation of ATP under aerobic condition. Together with the observations of a significant upregulation of KHK expression in response to fructose, these results indicate that in N2a cells activation of fructolysis with the subsequent generation of pyruvate may account for the increase in ATP production in response to fructose. The significance of KHK in the fructose-induced metabolic syndrome was demonstrated in a recent study in which the syndrome was prevented in mice lacking the KHK isoforms [38]. However, whether the same mechanism is involved in the HFD-induced ATP production in RVLM and manifest of hypertension await further elucidation. This can be further studied by the lentiviral transfection of KHK shRNA to knocking down KHK expression in RVLM.

While fructose shares structure similarity with glucose, they are metabolized in completely different ways and utilize different GluT transporters [39-42]. GluT5 is the primary transporter facilitating the diffusion of fructose across the cell membrane [40], whereas GluT2 prefers to transport glucose [41,42]. In addition, recent studies have shown that GluT2 also exhibits an important role in the absorption and transport of fructose [41,42]. In the current study, we found that both GluT2 and GluT5 are upregulated in the N2a cells following fructose exposure. In view that levels of the intermediate metabolite of glucose, glucose-6-phosphate, in the cells were not affected by high fructose, we reason that the increases in both GluT2 and GluT5 transporters may contribute to an increase in absorption and transport to the N2a cells of fructose, which is in turn metabolized to pyruvate via fructolytic process. Our results, at the same time, provide evidence to support the notion that fructose can be utilized by neurons as a carbon source of ATP production.

We provide direct evidence of a functional role of ATP in the N2a cells to promote neurotransmitter release. Not only fructose evoked a dose-dependent release of dopamine, manipulations that blocked the fructose-induced ATP production also blunted dopamine release. It is thus reasonable to postulate that exaggerated ATP generated following high fructose exposure may lead to activation of neurotransmission and the release of the neurotransmitter. ATP is known for its role in exocytosis for neurotransmitter release. ATP supply is indispensable for vesicle mobilization into the readily releasable pool [43,44]. In addition, following exocytosis, the rate of recovery of neurotransmitter release is also determined by vesicle retrieval from the plasma membrane, which is dependent on ATP supply [45].

We recognize that fructose may not satisfy the satiety center in the brain the way glucose does, and that food intake in the HFD group can be higher than that in the ND group [46]. This higher caloric intake may contribute to the harmful metabolic effects other than fructose per se [47]. In this regard, our data show similar daily food intake and the body weight in the ND and HFD animals. In addition, the HFD (3.6 Kcal/g) we used has a comparable calorie to that of the ND (4.8 Kcal/g). It is thus deemed unlikely the confounding effect of high caloric intake in the HFD group on the induced hypertension. We also recognize that endothelial dysfunction [48], activation of proinflammatory cytokines [49,50] and renal oxidative stress [51] are all engaged in pathogenesis of the metabolic syndrome and hypertension induced by the HFD.

It is unclear in the current study how fructose reaches RVLM following a long-term HFD. In this regard, fructose is capable to cross the blood–brain barrier [52]. The pericytes making close contacts with the cerebral microvascular endothelial cells are vital for viability and function of the blood–brain barrier [53]. It was recently reported [54] that a decline in the number of cerebral pericytes is responsible for glucose uptake and the pathological changes in the brain induced by hyperglycemia. In the central nervous system, astrocyte-neuron communication has recently been proposed as a potential mechanism participating in synaptic transmission [55]. Astrocytes are capable of generating and releasing ATP. Whether the results obtained from the *in vitro* N2a cells could be translated to explain the observations in the animal study, therefore, await further validation. In addition, the role of astrocytes on ATP production and activation of sympathetic premotor neurons in RVLM to the HFD also awaits further investigation.

## Conclusion

**Figure 8:**
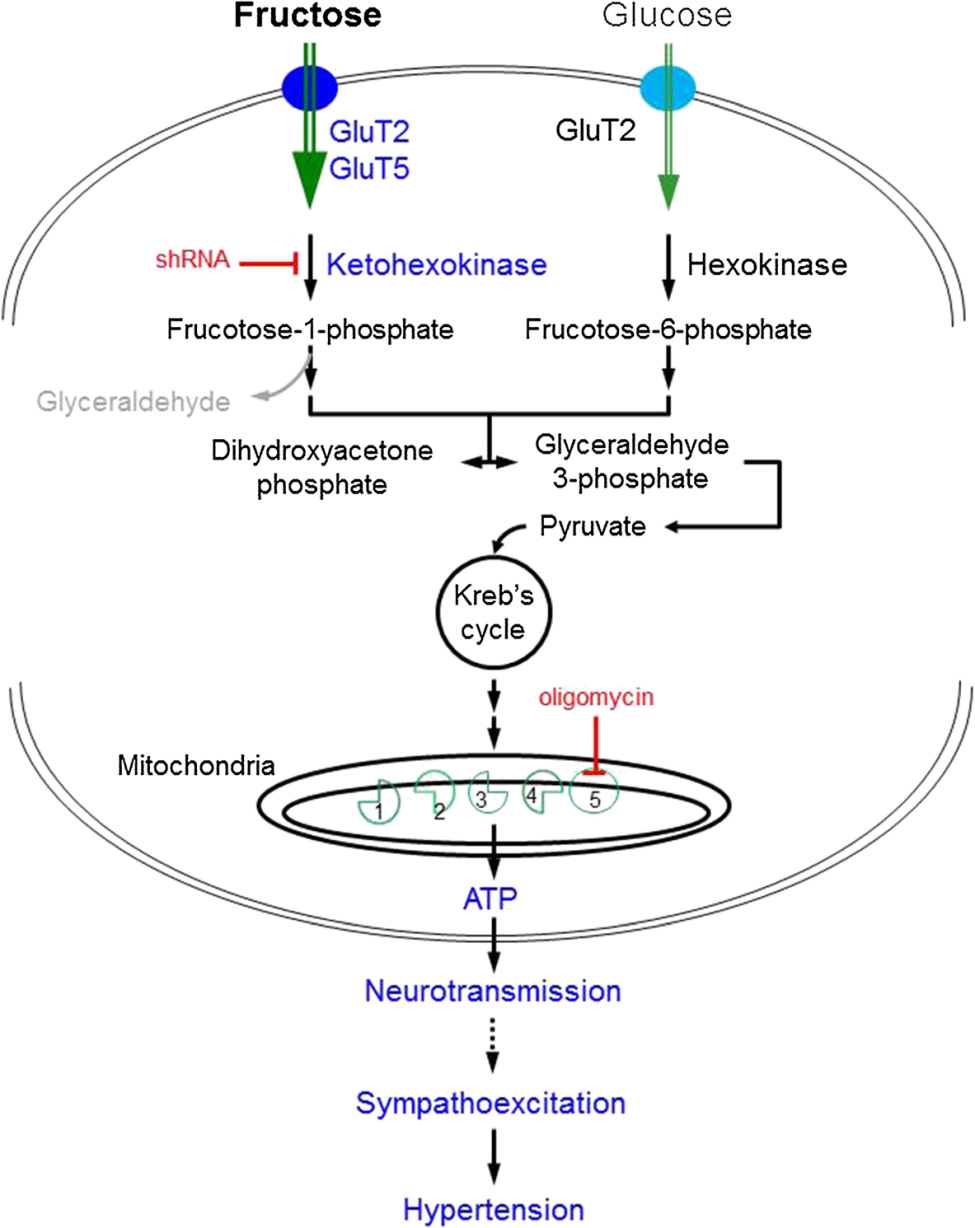
**Schematic illustrations showing potential mechanisms underlying the increase in cellular ATP production by high fructose.** In this scheme, high fructose upregulates both GluT2 and GluT5 expression, resulting in an increase in cellular uptake of fructose and subsequently a greater generation of pyruvate via activation of fructolysis. This high cellular level of pyruvate then enters the citric acid cycle as acetyl coenzyme to produce a greater amount of ATP via the mitochondrial respiratory chain complexes, resulting in an increase in the release of neurotransmitter. The shRNA gene silencing of the rate-limiting enzyme, ketohexokinase, blocks generation of pyruvate and the ATP synthase inhibitor, oligomycin, blunts the mitochondrial oxidative phosphorylation to prevent the high fructose-induced ATP generation.

The present study unveiled a potential novel mechanism underlying the sympathoexcitation and hypertension observed following the HFD fed, the increase in ATP production in the RVLM. We further demonstrated that, in a catecholaminergic neuronal cell line, fructose increases pyruvate and ATP production via upregulations of GluT2/5 and KHK, resulting in an increase in neurotransmitter (e.g., dopamine) release. Given the roles of GluT2/5 in fructose uptake and KHK in fructolysis, we proposed that, at a cellular level, an increase in GluT2/5 expression may cause an increase in cellular uptake of fructose and subsequently a greater generation of pyruvate via activation of fructolysis. This high cellular level of pyruvate then enters the citric acid cycle as acetyl coenzyme to produce a greater amount of ATP via the mitochondrial respiratory chain complexes, resulting in an increase in the release of neurotransmitter (Figure 8). This ATP-dependent increase in neurotransmitter release is further postulated to be involved in sympathoexcitation and hypertension induced by the HFD.

## Abbreviations

ATP: Adenosine-5’-triphosphate; DAPI: 4’,6-diamidino-2-phenylindole; 2-DG: 2-deoxyglucose; 2-DG6P: 2- deoxyglucose 6-phosphate; GluT: Glucose transporter; HFD: High fructose diet; KHK: Ketohexokinase; LF: Low frequency; RVLM: Rostral ventrolateral medulla; MTS: 3-(4,5-dimethylthiazol-2-yl)-5-(3-carboxymethoxyphenyl) -2-(4-sulfophenyl)-2H- tetrazolium; N2a: Neuro 2a; ND: Normal diet; SAP: Systolic arterial pressure; shRNA: Short hairpin RNA; WKY: Wistar-Kyoto.

## Competing interests

The authors declare that they have no competing interests.

## Authors’ contributions

KLHW conceived and designed the study as well as analyzed and interpreted the data, and wrote the manuscript. KLHW, CWW, and CYH performed experiments for data acquisition and performed the statistical analysis. JYHC was involved in data interpretation and revising the manuscript critically for important intellectual content. The authors have read and approved the final version of this manuscript.

## Authors’ information

KLHW is currently a principle investigator in the Center for Translational Research in Biomedical Sciences in Kaohsiung Chang Gung Memorial Hospital.

CWW is a postdoctoral fellow in the Center for Translational Research in Biomedical Sciences in Kaohsiung Chang Gung Memorial Hospital.

CYH is currently an experimental assistant in the Center for Translational Research in Biomedical Sciences in Kaohsiung Chang Gung Memorial Hospital.

JYHC is the Chair professor in the Center for Translational Research in Biomedical Sciences, and Director of Department of Medical Research in Kaohsiung Chang Gung Memorial Hospital. She is currently the President of the Federation of Asian and Pacific Physiological Societies and the Vice President of International Union of Physiological Sciences.

## Supplementary Material

Additional file 1: Figure S1 ATP contents in the hippocampus. Tissue levels of ATP in the hippocampus detected in week 12 after the ND or HFD feeding. Values are mean ± SEM (n = 8 to 12 animals in each experimental group). No significant difference between the two groups in the student *t* test. Click here for file

Additional file 2: Figure S2 Tissue ROS level in the RVLM of rat fed the ND or HFD. Percentage of the DHE-positive cells in the RVLM detected in week 12 after the ND or HFD. Values are mean ± SEM (n = 8 to 12 animals in each experimental group). No significant difference between the two groups in the student *t* test. Click here for file
