# Supplementary figures and images for: An increase in adenosine-5’-triphosphate (ATP) content in rostral ventrolateral medulla is engaged in the high fructose diet-induced hypertension
Source: J Biomed Sci. 2014 Jan 27;21(1):8. doi: 10.1186/1423-0127-21-8 (PMC3913325; doi:10.1186/1423-0127-21-8)

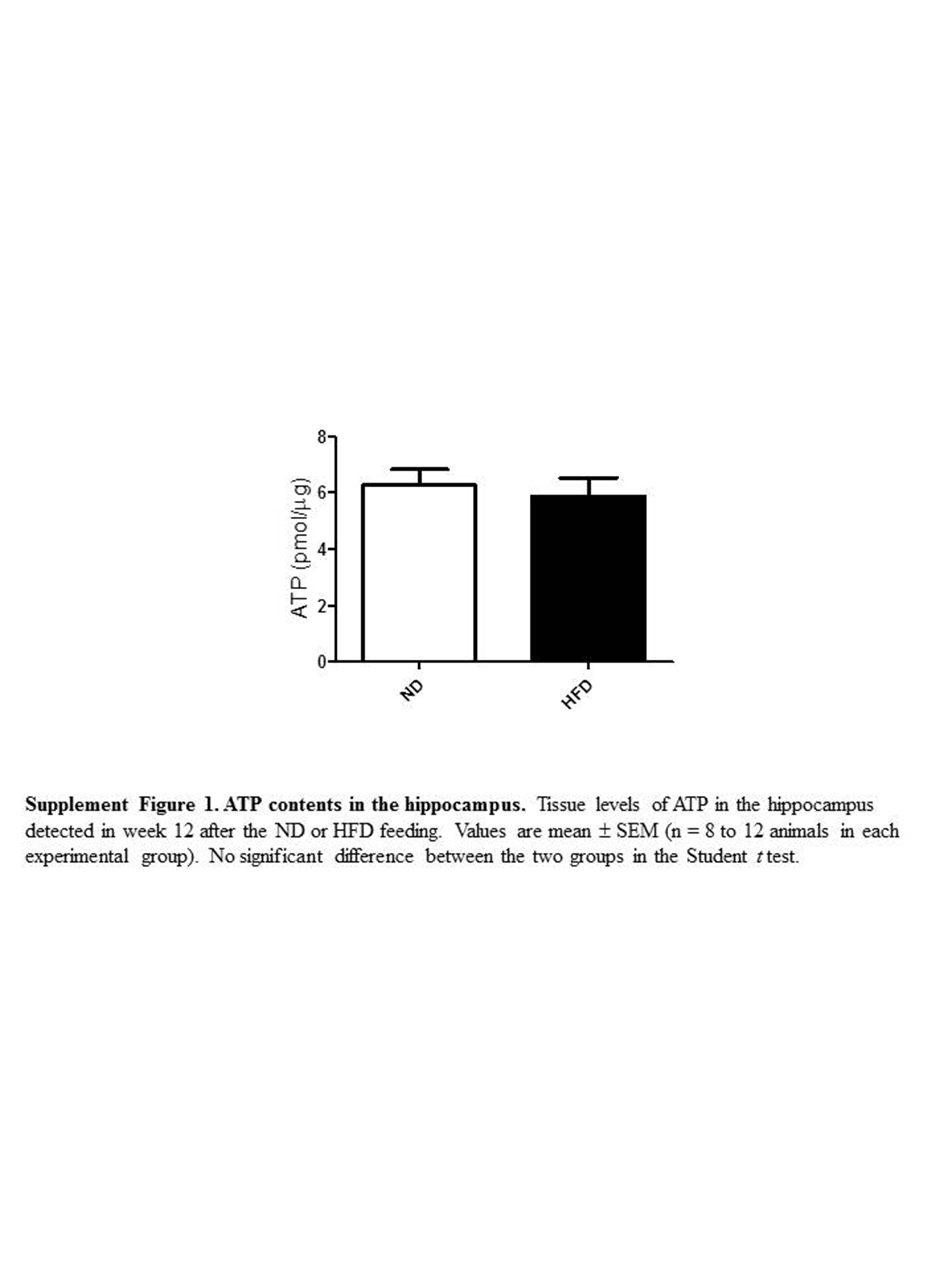

Supplement: Additional file 1: Figure S1 — ATP contents in the hippocampus. Tissue levels of ATP in the hippocampus detected in week 12 after the ND or HFD feeding. Values are mean ± SEM (n = 8 to 12 animals in each experimental group). No significant difference between the two groups in the student t test. [file 1423-0127-21-8-S1.jpeg]

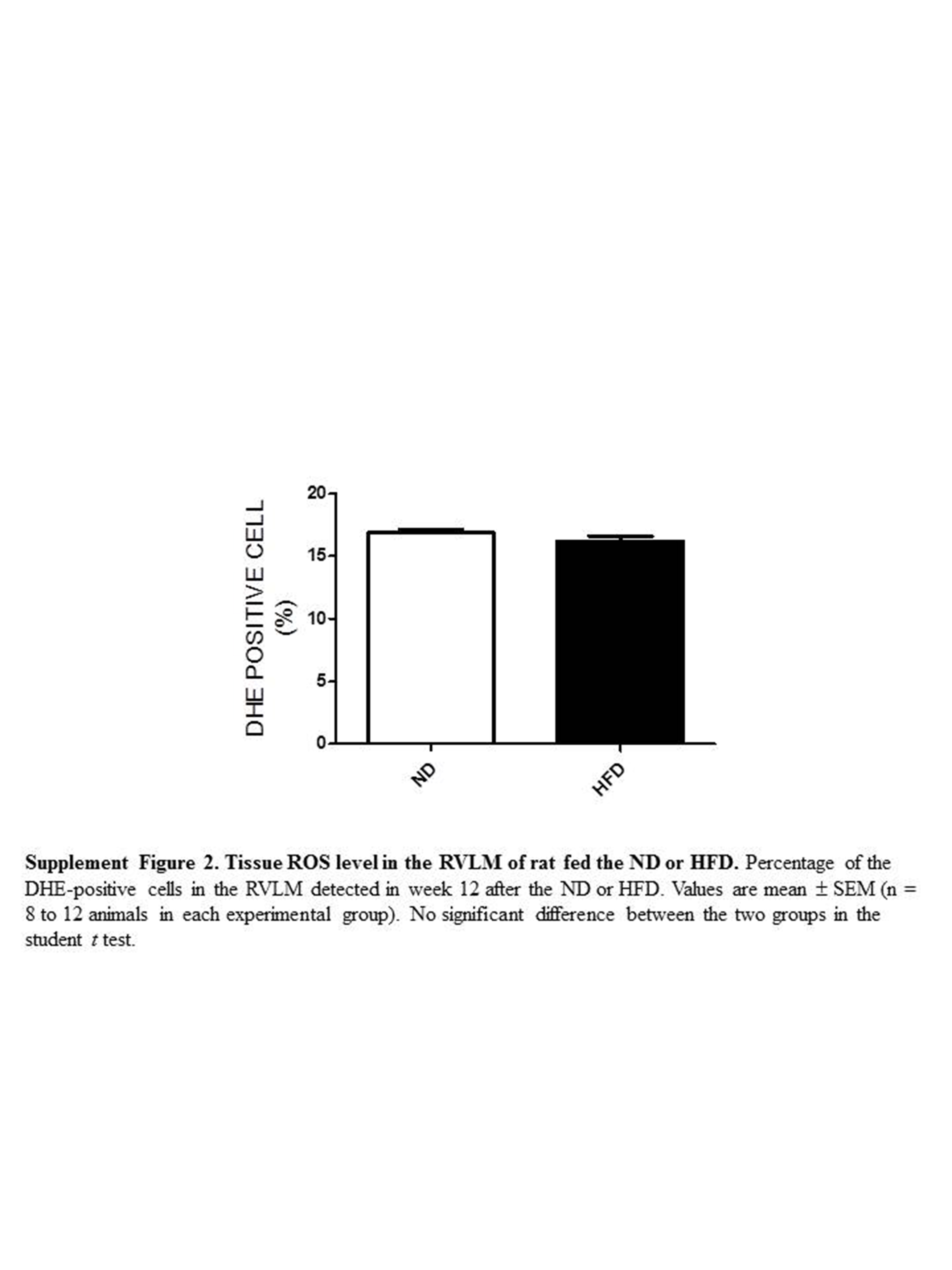

Supplement: Additional file 2: Figure S2 — Tissue ROS level in the RVLM of rat fed the ND or HFD. Percentage of the DHE-positive cells in the RVLM detected in week 12 after the ND or HFD. Values are mean ± SEM (n = 8 to 12 animals in each experimental group). No significant difference between the two groups in the student t test. [file 1423-0127-21-8-S2.jpeg]
